# Numerical Study of Double-Layered Microchannel Heat Sinks with Different Cross-Sectional Shapes

**DOI:** 10.3390/e21010016

**Published:** 2018-12-25

**Authors:** Daxiang Deng, Guang Pi, Weixun Zhang, Peng Wang, Ting Fu

**Affiliations:** 1 Department of Mechanical & Electrical Engineering, Xiamen University, Xiamen 361005, China; 2 Shenzhen Research Institute of Xiamen University, Shenzhen 518000, China; 3 Tianjin Key Laboratory for Civil Aircraft Airworthiness and Maintenance Airworthiness and Maintenance, Civil Aviation University of China, Tianjin 300300, China; 4 Hubei Key Laboratory of Mechanical Transmission and Manufacturing Engineering in Wuhan University of Science and Technology, Wuhan 430000, China

**Keywords:** double-layered microchannel heat sinks, microchannel shape, numerical simulation, thermal resistance

## Abstract

This work numerically studies the thermal and hydraulic performance of double-layered microchannel heat sinks (DL-MCHS) for their application in the cooling of high heat flux microelectronic devices. The superiority of double-layered microchannel heat sinks was assessed by a comparison with a single-layered microchannel heat sink (SL-MCHS) with the same triangular microchannels. Five DL-MCHSs with different cross-sectional shapes—triangular, rectangular, trapezoidal, circular and reentrant Ω-shaped—were explored and compared. The results showed that DL-MCHS decreased wall temperatures and thermal resistance considerably, induced much more uniform wall temperature distribution, and reduced the pressure drop and pumping power in comparison with SL-MCHS. The DL-MCHS with trapezoidal microchannels performed the worst with regard to thermal resistance, pressure drop, and pumping power. The DL-MCHS with rectangular microchannels produced the best overall thermal performance and seemed to be the optimum when thermal performance was the prime concern. Nevertheless, the DL-MCHS with reentrant Ω-shaped microchannels should be selected when pumping power consumption was the most important consideration.

## 1. Introduction

With the rapid development of microelectronic devices, the local heat flux inside has so far increased to more than 300 W/cm^2^ [1], which is far beyond the heat dissipation limit of air cooling schemes. Heat removal is thus vital for the safe and steady operation of microelectronic devices. Microchannel heat sinks, which were proposed by Tuckerman and Pease [2] in 1981, have been recognized to be an efficient means to dissipate high heat flux. Due to its high surface area to volume ratio, large heat transfer coefficient, and small coolant inventory, the microchannel heat sink has been used in recent years as a high-performance compact cooling method in thermal dissipation applications of very-large-scale integrated (VLSI) circuits, microelectromechanical systems, and high power laser diode arrays [3,4,5]. 

In microchannel heat sinks, coolant flows in parallel microchannels from inlet to outlet in a single direction. Coolant temperature increases along the stream-wise direction and results in a poor heat exchange process between the coolant and microchannel wall. As a result, the bottom wall temperatures of the heat sink increase along the flow length. Non-uniform wall temperature distribution induces undesirable thermal stresses in microelectronic devices and hence reduces reliability and shortens the lifetime of microelectronic devices. To address this issue, Vafai and Zhu [6] proposed the concept of a double-layered microchannel heat sink (DL-MCHS) in which two single-layered microchannel heat sinks (SL-MCHS) are stacked one on top of the other. It was found that the streamwise temperature rise was substantially reduced for DL-MCHS and that the pressure drop was also smaller than that of SL-MCHS. Motivated by these promising results, extensive studies have been subsequently conducted to explore the thermal and fluid properties of DL-MCHS [7,8,9,10,11,12,13,14,15,16,17,18,19,20,21]. Early studies mainly focused on the effectiveness verification of the DL-MCHS design. Chong et al. [7] numerically compared single-layered and double-layered counter flow microchannel heat sinks with rectangular shapes by employing a thermal resistance network and 3-D computational fluid dynamics (CFD) simulations. The superiority of DL-MCHS was demonstrated again. Cheng [8] found in numerical simulations that the thermal resistance of DL-MCHS can be reduced by 47.7% compared to that of a SL-MCHS. 

Following this, research attention has been paid to the optimization design of geometric parameters and the flow arrangement of DL-MCHS. Wei et al. [9] fabricated a stacked microchannel heat sink using silicon micromachining techniques. The effects of flow direction and flow rate ratio in each microchannel layer were explored both experimentally and numerically. Thermal resistance as low as 0.09 °C/(W∙cm^2^) was obtained for a stacked microchannel heat sink with two layers of microchannels. The counter-flow arrangement provided better wall temperature uniformity whereas the parallel flow had the best performance in reducing the peak temperature. Hung et al. [10] identified that several dominant parameters—substrate materials, coolants, and geometric parameters such as channel number, channel width ratio, channel aspect ratio, substrate thickness, and pumping power—play a notable role in the temperature distribution, pressure drop, and thermal resistance of DL-MCHS with rectangular microchannels. They also provided an optimization procedure for the geometric parameters of DL-MCHS via a simplified conjugate-gradient method and a three-dimensional fluid flow and heat transfer model [11]. Xie et al. [12] explored the layout effect of parallel-flow and counter-flow for inlet and outlet flow directions on the thermal performance of a DL-MCHS with rectangular microchannels. The results showed that the parallel-flow layout presented better heat dissipation when the flow rate was limited to a low value whereas the counter-flow layout did better in high flow rate cases. The effect of the height of the upper-branch and lower-branch channels on the thermal performance of DL-MCHS has also been assessed. Lin et al. [13] conducted optimization studies on DL-MCHS with rectangular microchannels to search for a minimum of the thermal resistance. Six design variables, including channel number, vertical rib width, bottom channel height, thicknesses of two horizontal ribs, and coolant velocity in the bottom channel were simultaneously optimized by a three-dimensional solid-fluid conjugated model coupled with a simplified conjugate-gradient. Leng et al. [14] optimized channel number, channel width, bottom channel height, and bottom coolant inlet velocity to improve bottom wall temperature uniformity and to reduce the overall thermal resistance for DL-MCHS with rectangular microchannels at constant pumping powers.

Recently, DL-MCHSs with advanced configurations or flow passage design have been developed to enhance thermal and fluid flow characteristics. The research groups of Wang et al. have developed improved designs of double-layered rectangular microchannel heat sinks with truncated top channels [17] and porous fins [18] to promote the thermal and hydraulic performance of DL-MCHS. Such new designs of DL-MCHS have been found to reduce thermal resistance and pumping power considerably. Osanloo et al. [19] and Wong et al. [20] developed DL-MCHS with tapered microchannels or channel contraction. Higher thermal performance was achieved for such improved DL-MCHS as compared to that with the conventional design of straight channels, but pumping power was increased. Zhai et al. [21] have developed double-layered microchannel heat sinks with cavities and ribs in the rectangular microchannel flow passages. The comparisons among them and conventional DL-MCHSs and single-layered micro heat sinks with simple structures revealed that DL-MCHS with cavities and ribs showed better heat transfer characteristics under the same volumetric flow rate. 

In previous reports of double-layered microchannel heat sinks, parallel microchannels with conventional rectangular shapes have generally been employed, and the geometric parameters of rectangular microchannels, such as channel number, channel width, channel height and channel aspect ratio, have been focused on. Nevertheless, except for the studies of DL-MCHS with trapezoidal and rectangular shapes in Sharma et al. [22] and for that including DL-MCHS with boot, diamond, hexagonal, pentagonal, rectangular, rectangular wedge, and triangular shapes in Kulkarni et al. [23], information about DL-MCHS with different cross-sectional shapes is still far from sufficient. To address this, we in this study developed five double-layered microchannel heat sinks with different microchannel cross-sectional shapes, i.e., rectangular, triangular, trapezoidal, circular, and reentrant Ω-shaped. The thermal and hydraulic performance of these five DL-MCHSs were explored by numerical simulations. Moreover, a comparison between DL-MCHS and SL-MCHS was also conducted using the triangular microchannels. This study sheds some light on the design of double-layered microchannel heat sinks and is believed to be of practical importance. 

## 2. Model Description 

**Figure 1:**
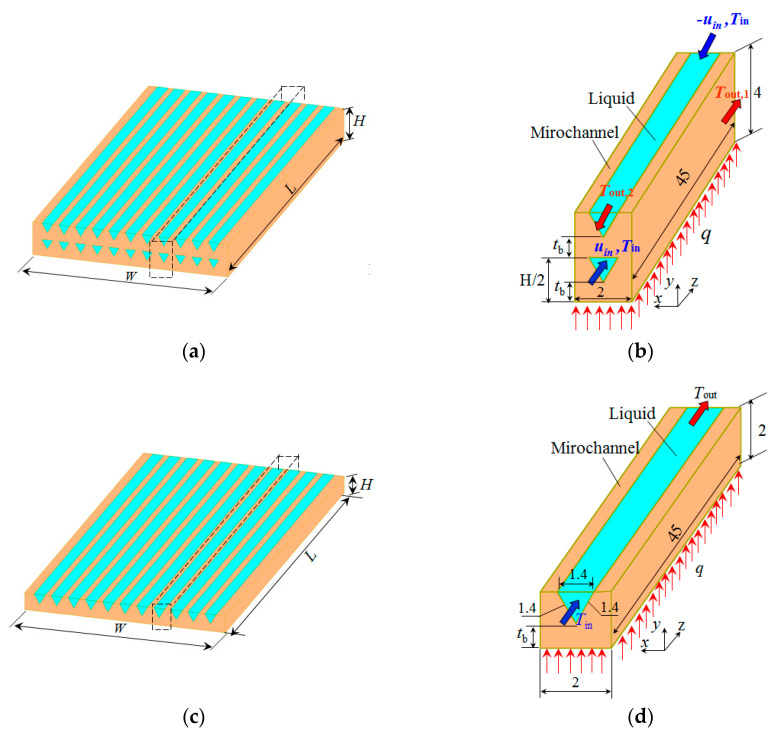
Schematic of double-layered and single-layered micro-channel heat sinks: (**a**) a typical DL-MCHS; (**b**) a unit cell for the computational domain of DL-MCHS; (**c**) a typical SL-MCHS; (**d**) a unit cell for the computational domain of SL-MCHS.

**Figure 2:**
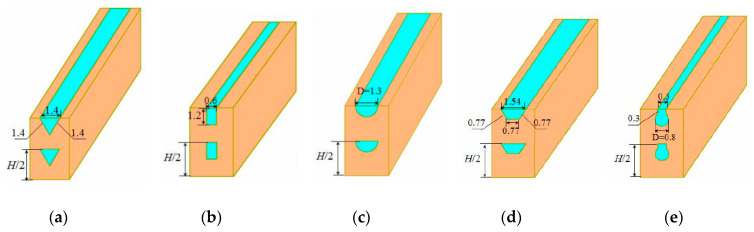
Schematic of the DL-MCHS with different microchannel cross-sectional shapes: (**a**) triangular; (**b**) rectangular; (**c**) circular; (**d**) trapezoidal; (**e**) reentrant Ω-shaped.

The double-layered microchannel heat sink is illustrated in Figure 1a. Two layers of microchannels with the same cross-sectional shape and geometric dimensions are stacked together. Due to the symmetric and periodic arrangement of microchannels in double-layered microchannel heat sinks, a unit cell containing a microchannel is chosen as the computational domain, as shown in Figure 1. Five DL-MCHS with different microchannel cross-sectional shapes—triangular, rectangular, circular, trapezoidal and reentrant Ω-shaped—were numerically studied to explore the effect of microchannel cross-sectional shape on the performance of DL-MCHS. The microchannels were designed to have nearly the same hydraulic diameter, as shown in Table 1. The geometric dimensions of each microchannel are shown in Figure 2. The reentrant Ω-shaped microchannels, which feature large circular cavities inside and an exit narrow slot at the top of the cross section, have been found to show good heat transfer performance in convective flow in the single-layered microchannel heat sinks [24]. The thermal and fluid flow behaviors of these microchannels in double-layered microchannel heat sinks are thus explored in this study. The counter flow arrangement of two layers of DL-MCHS was adopted following the findings of many previous reports [9,12,25], i.e., the fluid flows through the lower channel in the positive *Z* direction and through the upper channel in the negative *Z* direction. For comparison, a single-layered microchannel heat sink with triangular shapes was also studied. The unit cell of the SL-MCHS is of the same width of 2 mm and the same length of 45 mm but had a height half of that of the DL-MCHS. All the geometric parameters of these samples are shown in Figure 2 and Table 1. Uniform heat power *q* is supplied to the bottom of the heat sink to simulate a heating element such as micro-processor chips. The microchannel heat sinks are made of copper and the coolant used is water.

## 3. Numerical Methods

### 3.1. Governing Equations and Models

A three-dimensional solid–fluid conjugate model is utilized to determine the thermal and hydraulic performance of microchannel heat sinks. The following assumptions are taken into account to simplify the analysis: (1) the flow is three-dimensional, incompressive, laminar, and in steady-state; (2) the effect of gravity forces are considered; (3) the fluid thermophysical properties are temperature-dependent; (4) radiation heat transfer is negligible; (5) all solid walls of the channel are no-slip and impermeable; (6) viscous dissipation effects are negligible.

According to the above assumptions, the governing equations can be expressed as follows: 

Continuity equation:

(1) $$\nabla \cdot (\rho \vec{v})=0$$

Momentum equation:

(2) $$\nabla \cdot (\rho \vec{v}\vec{v})=-\nabla P+\nabla \cdot (\mu \nabla \vec{v})$$

Energy equation for the liquid:

(3) $$\nabla \cdot (\rho \vec{v}c_{p}T)=\nabla \cdot (k_{f}\nabla T)$$

Energy equation for the solid microchannel:

(4) $$\nabla \cdot (k_{m}\nabla T)=0$$

### 3.2. Boundary Conditions 

The boundary conditions are provided as follows. In the solid region, a uniform heat flux (12.67 W/cm^2^) was applied to the bottom surface of the unit cell. The top surface above the microchannel fins and liquid is set to be adiabatic. A symmetry wall boundary condition is applied on both outer lateral planes of the computational domain. In the fluid region, a fully developed and uniform liquid velocity with two values (0.0625 m/s and 0.125 m/s) and a constant inlet temperature (T_in_ = 33 °C or 306 K) is applied to both inlets of the upper and lower layers of microchannels in DL-MCHS. At the outlet of the microchannels, a pressure outlet boundary condition is specified as the atmospheric pressure. 

### 3.3. Numerical Methods 

**Figure 3:**
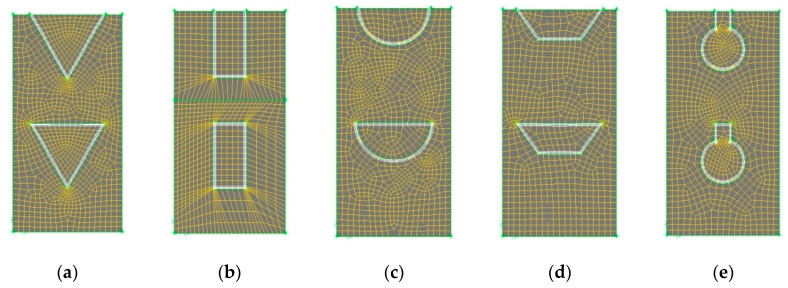
Illustration of grid generations for DL-TRI with different microchannel cross-sectional shapes: (**a**) triangular; (**b**) rectangular; (**c**) circular; (**d**) trapezoidal; (**e**) reentrant Ω-shaped.

The computations are carried out in a finite-volume based computational fluid dynamics (CFD) software package, FLUENT. The simulation domains are generated in Gambit v2.3. A second-order upwind scheme is used to discretize the convective term and a QUICK scheme is used for discretization of the diffusion term. The coupling between pressure and velocity is implemented by a SIMPLE algorithm. The solutions are thought to be converged when the normalized residuals are less than 10^−3^ for the flow equations and 10^−7^ for the energy equations. Unstructured grids with finer meshes near the fluid-solid wall regions are utilized, as illustrated in Figure 3. 

Grid sensitivity analysis was performed to check the independence of grids. The variations in pressure drop from inlet to outlet of the heat sink were chosen for the evaluation of grid dependence. Three non-uniform grid systems with denser grid clustering near the wall were employed with 0.204, 0.412, and 0.628 million grids, respectively. The deviations in pressure drop using 0.204 and 0.412 million grids as compared to that using 0.628 million grids were 5.6% and 1.1%, respectively. Excessive refinement of the grids seemed to provide no improvement to the computational results. Therefore, to save computing time and to maintain accuracy of the computed results, a grid number of 0.628 million was finally chosen for the simulations. 

### 3.4. Data Reduction

When the temperature and pressure drop results are obtained by numerical simulation, they could be processed to calculate the thermal and hydraulic characteristics.

The total thermal resistance of microchannel heat sinks is defined as [9,10]

(5) $$R_{t}=\frac{T_{\max}-T_{in}}{q}$$

where *T*_max_ is the maximum temperature of the microchannel base, *T*_in_ is the inlet coolant temperature, and *q* is the heat flow carried away by the coolant. 

Pumping power of the microchannel heat sink, *P*, is defined as

(6) $$P=\Delta p_{u}\cdot u_{u}\cdot A_{c,u}+\Delta p_{d}\cdot u_{d}\cdot A_{c,b}$$

where Δp is the pressure drop of the coolant between the microchannel inlet and outlet of the upper and lower layers,u is the inlet flow velocity, and *A*_c_ is the cross-sectional area of a microchannel. The subscripts “*u*” and “*d*” represent the upper and down/lower channels of DL-MCHS, respectively. 

The maximum temperature rise of $\Delta T_{w,\max}$ is determined by the temperature difference between the maximum and minimum wall temperatures in the microchannels. It is utilized to assess the wall temperature uniformity. 

### 3.5. Validation of Models and Methods

To validate the aforementioned numerical models and methods, a simulation of SL-MCHS with triangular microchannels was first conducted. The obtained fluid temperature differences from microchannel inlet to outlet with different inlet velocities were compared with the theoretical values predicted by the energy balance equations using [25]

(7) $$\Delta T=T_{out}-T_{in}=\frac{q}{\rho c_{p}\dot{V}}$$

It was found that the maximum relative deviation between the numerical results and theoretical predictions in the simulated velocity range was less than 3.5%. This suggests that the present numerical models and methods are reliable for the determination of the thermal and hydraulic performance of microchannel heat sinks. 

## 4. Results and Discussion

### 4.1. Heat Transfer Enhancement and Pressure Drop Reduction of DL-MCHS

**Figure 4:**
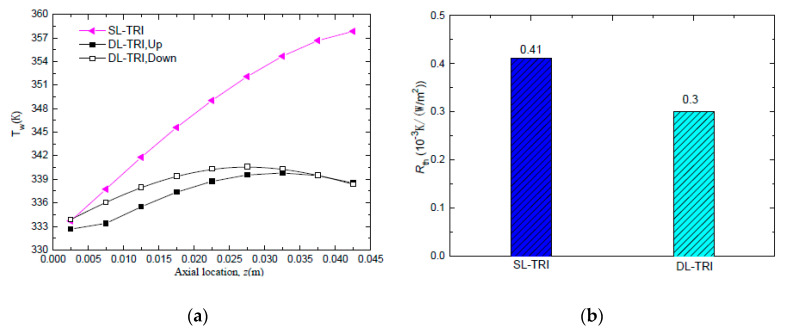
Comparison of wall temperature distribution and thermal resistance between SL-TRI and DL-TRI with the same triangular shape: (**a**) wall temperature distribution of the microchannel bottom surface along the axial flow direction; (**b**) thermal resistance.

To validate the superiority of DL-MCHS, a DL-MCHS sample with triangular shape (DL-TRI) was compared with a single-layered microchannel heat sink with the same shape (SL-TRI). As the flow area of double-layered microchannels was twice that of single-layered microchannels, the inlet velocity of the DL-TRI was set to be half of that of the corresponding single-layered microchannels at the same volumetric flow rate, i.e., 0.0625 m/s for DL-TRI, and 0.125 m/s for SL-TRI. Figure 4a shows the wall temperature distributions along the axial flow length for both the upper layer and lower layer of the DL-TRI and SL-TRI samples. It is clear that the DL-MCHS decreased the wall temperature rise significantly and presented much more uniform wall temperature distributions along the stream-wise direction. The maximum wall temperature of the microchannel bottom surface decreased from 358 K in SL-TRI to 340–341 K in DL-TRI. In addition, the maximum wall temperature difference was decreased by 72% for DL-TRI, that is, it decreased from 24 ℃ in SL-TRI to 7 ℃ in DL-TRI. A much more uniform wall temperature distribution in the double layer layout of microchannels helps to reduce thermal stresses caused by the temperature difference and facilitates improvement of the reliability of microelectronic devices.

**Figure 5:**
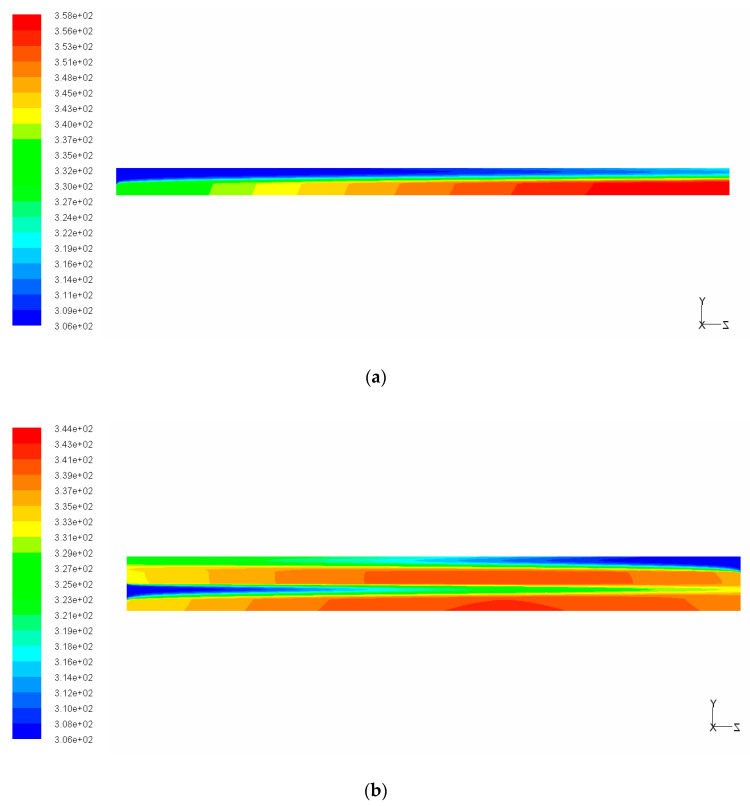
Temperature contours of the middle plane along the axial flow direction of SL-TRI and DL-TRI with the same triangular shape: (**a**) SL-TRI; (**b**) DL-TRI.

From Figure 4a, it can be noted that the wall temperature of SL-TRI increased monotonically from the microchannel inlet to the outlet, which can also be seen in the temperature contours in Figure 5a. This can be related to the deterioration of heat exchange between the wall and fluid with increasing fluid temperature along the flow length. Conversely, for the DL-TRI, the wall temperature first increased along the axial flow length in the upstream region, reached the maximum wall temperature in the middle to downstream region of the channels, and then tended to decrease in the downstream region. This can be also seen in the temperature contours of DL-TRI in Figure 5b. The counter-flow cooling effects of the coolant from the inlet of the upper microchannels of the DL-TRI contributed to decreasing wall temperatures in the downstream region. Thermal resistance was also reduced considerably from 0.41 × 10^−3^ K/(W/m^2^) in SL-TRI to 0.3 × 10^−3^ K/(W/m^2^) in DL-TRI, as shown in Figure 4b. The double-layered microchannel heat sinks presented a 27% reduction in thermal resistance, which is promising for the efficient cooling of high heat flux microelectronic devices.

**Figure 6:**
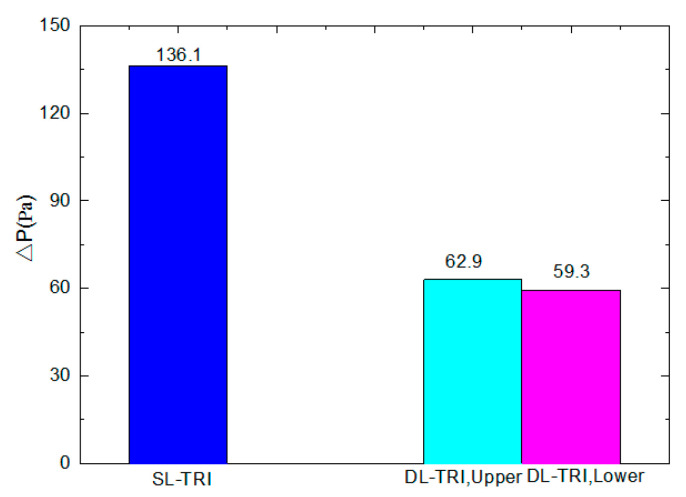
Comparison of pressure drop between SL-TRI and DL-TRI with the same triangular shape.

Figure 6 shows the comparison of pressure drop characteristics of SL-TRI and both the upper and lower layers of DL-TRI. Despite the same volumetric flow rate being applied for both microchannel heat sinks, and the inlet flow velocity of SL-TRI being equal to the sum of both the upper and lower layers of DL-TRI, the SL-TRI still presented a larger ΔP than the sum of both the upper and lower layers of DL-TRI (122.2 Pa). The above results are consistent with those of previous reports [6,12], suggesting that the DL-TRI far outperformed its single-layered counterpart in the reduction of both pressure drop and pumping power. In this regard, the double-layered microchannel heat sink showed its merits in both heat transfer enhancement and pressure drop reduction compared to the single-layered microchannel heat sink.

### 4.2. Effects of Microchannel Shape on the Performance of DL-MCHS

#### 4.2.1. Thermal Characteristics

**Figure 7:**
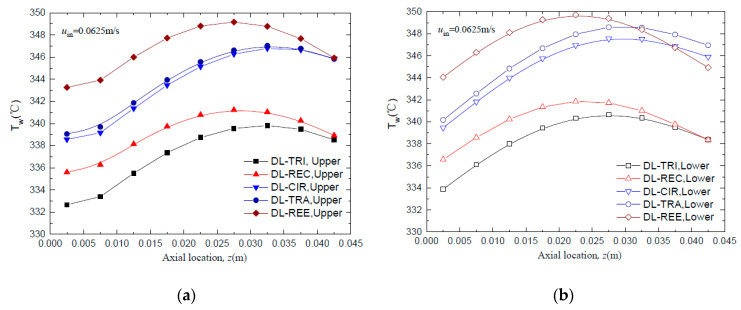
Comparison of wall temperature distributions along the axial flow length for all five DL-MCHSs with flow velocities of 0.0625m/s: (**a**) upper layer; (**b**) lower layer.

**Figure 8:**
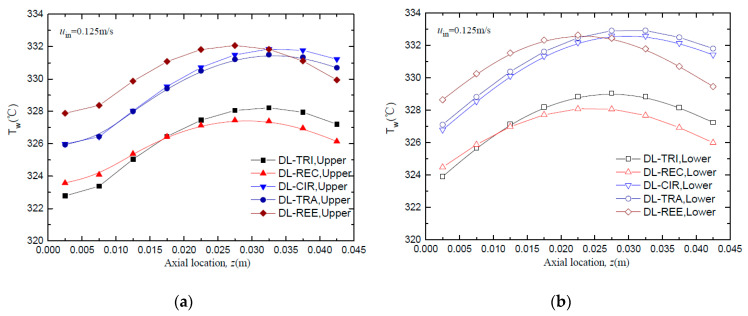
Comparison of wall temperature distributions along the axial flow length of all five DL-MCHSs with flow velocities of 0.125m/s: (**a**) upper layer; (**b**) lower layer.

**Figure 9:**
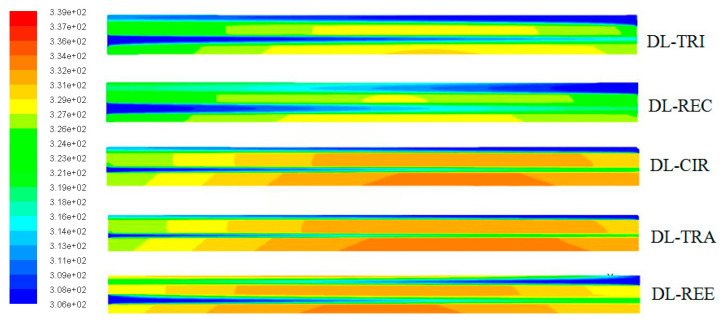
Temperature contours in the middle plane along the axial flow direction of all five DL-MCHSs.

**Figure 10:**
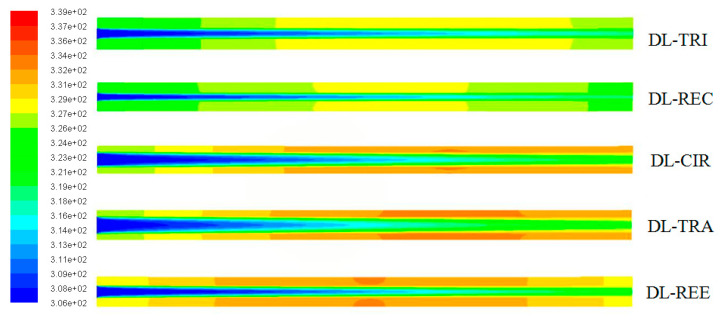
Temperature contours in the middle height plane of microchannels in the lower layer of the five DL-MCHSs.

The wall temperature distributions along the axial flow length for all five DL-MCHS with different cross-sectional shapes are shown in Figure 7 and Figure 8 at flow velocities of 0.0625 m/s and 0.125 m/s, respectively. All DL-MCHS samples first presented an increase in the *T*_w_, then reached the plateau in the middle to downstream region of the channels, and then tended to decrease in the downstream region. For both inlet velocity cases, it is clear that the DL-TRI with triangular microchannels and the DL-REC with rectangular microchannels showed notably lower wall temperatures in both the upper and lower layers of microchannel bases in comparison to the other three samples. These two samples were of large microchannel height (about 1.2 mm), which induced the small thickness of the bottom wall, as shown in Table 1. As uniform heat fluxes were imposed on the bottom surface of the samples, heat was transported vertically in the upper direction by heat conduction. The DL-TRI and DL-REC with small thicknesses of bottom walls facilitated the reduction of the length of heat conduction, and thus reduced the conducted thermal resistance in the solid bases [9]. The heat was dissipated much quicker by the fluid in the microchannels, and smaller wall temperatures can be noted. This can be also seen in the temperature contours of the DL-MCHS samples in Figure 9 and Figure 10.

**Figure 11:**
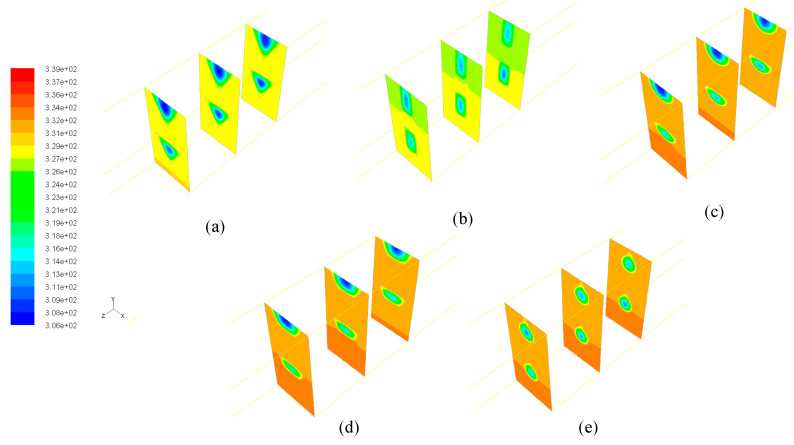
Temperature contours in three cross-sectional planes (z = 20, 22.5, and 25 mm) in the middle regions of five DL-MCHSs with flow velocities of 0.125m/s: (**a**) DL-TRI; (**b**) DL-REC; (**c**) DL-CIR; (**d**) DL-TRA; and (**e**) DL-REE.

For the other three DL-CIR, DL-TRA, and DL-REE samples, the *T*_w_ of DL-CIR was close to those of the DL-TRA in general. The DL-REE with reentrant Ω-shaped microchannels showed the highest wall temperatures in the upstream to middle regions. Nevertheless, the DL-REE presented a more rapid decrease in the *T*_w_ in the downstream region, which induced lower wall temperatures than for DL-CIR and DL-TRA in this region. For the DL-REE with reentrant Ω-shaped microchannels, as the main flow was located inside the large circular portion of the reentrant cavities, the heat exchange between the hot wall and fluid mainly occurred in this circular cavity area. Since the vertical distances from the microchannel bottom surface to the circular cavity of DL-REE were smaller than those of DL-CIR and DL-TRA, the conducted thermal resistance was reduced for DL-REE. Therefore, more heat was dissipated from the microchannel solid wall to the fluid in the reentrant Ω-shaped microchannels, and smaller wall temperatures were induced in the downstream regions. Such behaviors can also be seen in the temperature contours in Figure 9, Figure 10 and Figure 11. The DL-CIR and DL-TRA showed more portions of high wall temperature regions in the middle to downstream areas, which is especially notable in the microchannel base of the lower layer. For the DL-REE, however, significantly fewer portions of high wall temperature regions can be seen only in the middle region.

**Figure 12:**
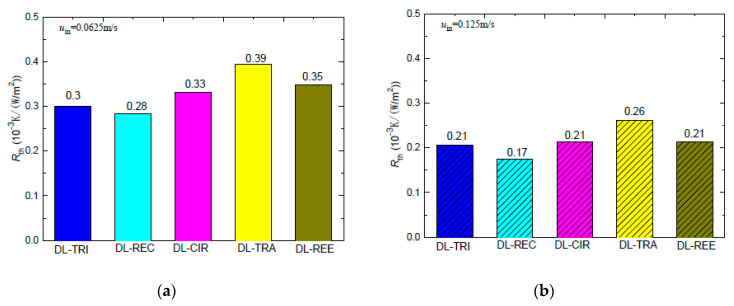
Thermal resistance of five DL-MCHSs for test cases of: (**a**) 0.0625 m/s; (**b**) 0.125 m/s.

Table 2 lists the maximum wall temperature differences along the flow length for DL-REE. It was found that DL-CIR and DL-TRA presented the worst wall temperature uniformity, followed by DL-TRI and DL-REE. The DL-REC with rectangular microchannels presented the most uniform temperature distributions, indicating that it is more suitable for reducing thermal stresses in the cooling of microelectronic devices. The total thermal resistance of the five DL-MCHS samples is shown in Figure 12. For both flow velocities cases, the DL-TRA with trapezoidal microchannels showed the largest thermal resistance, followed by DL-CIR and DL-REE. This can be related to the fact that these three samples presented large wall temperatures as discussed above. The DL-REC with rectangular microchannels showed the smallest thermal resistance. The rectangular microchannels with flat bottom walls and large channel height seemed to help in the heat transfer process of coolants and presented the best overall thermal performance.

#### 4.2.2. Pressure Drop and Pumping Power

**Figure 13:**
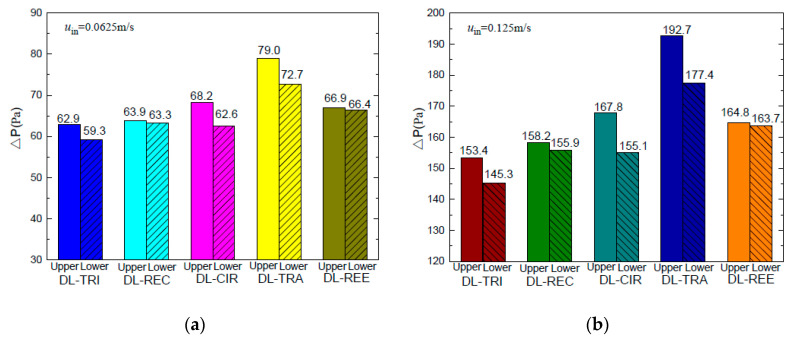
Pressure drop of five DL-MCHSs in the test cases of: (**a**) 0.0625 m/s; (**b**) 0.125m/s.

The pressure drop characteristics of both layers in the five double-layered microchannel heat sinks are shown in Figure 13a,b. The upper layer presented larger pressure drops than the lower layer for all five DL-MCHSs. This was especially notable in the DL-TRA with trapezoidal microchannels. As the microchannel wall and fluid temperatures in the bottom base were larger than in the upper layer, the fluid viscosity in the lower layer decreased with elevation of fluid temperatures. This resulted in the reduction of viscous forces and thus induced a smaller pressure drop in the lower layer.

**Figure 14:**
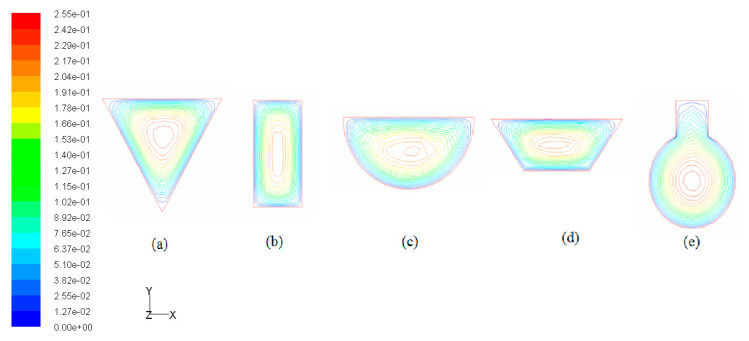
Velocity streamlines in the cross-sectional planes of outlets in the lower layer of five DL-MCHSs with a flow velocity of 0.125 m/s: (**a**) DL-TRI; (**b**) DL-REC; (**c**) DL-CIR; (**d**) DL-TRA; and (**e**) DL-REE.

**Figure 15:**
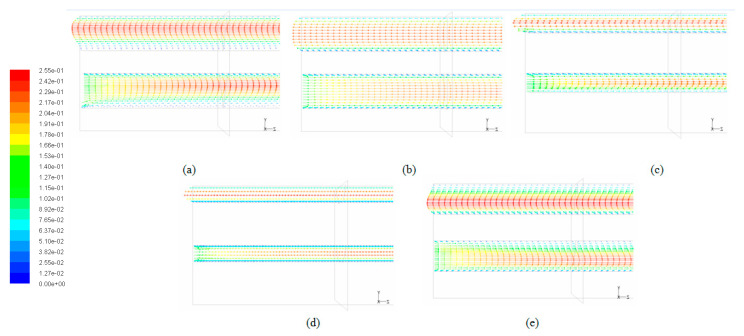
Velocity vectors in the middle *x* plane of five DL-MCHSs in the entrance regions (*z* = 0–5 mm) with a flow velocity of 0.125 m/s: (**a**) DL-TRI; (**b**) DL-REC; (**c**) DL-CIR; (**d**) DL-TRA; and (**e**) DL-REE.

When considering the sum of total pressure drops in both layers of the double-layered microchannel heat sinks, it can be found that the DL-TRA with trapezoidal microchannels induced the largest pressure drop, followed by DL-REE, DL-CIR, and DL-REC. The DL-TRI with triangular microchannels definitively presented the smallest pressure drop. The above trend can be related to the flow morphologies inside the microchannels with different shapes. Figure 14 illustrates the velocity streamlines in the cross-sectional plane of the outlet of the five DL-MCHSs, and Figure 15 shows the velocity streamlines along the middle *x* plane in the streamwise directions in the entrance regions (*z* = 0–5 mm). Since the DL-TRA and DL-CIR featured large width and small height in the flow passages, they played a confinement role in the development of flow streamlines in the vertical direction. The velocity magnitude quickly decreased in the vertical direction from the core region to the boundary layer due to the action of the molecular viscous force drag [26]. Therefore, due to the confinement of the trapezoidal and circular configurations, a large pressure drop can be obtained from the DL-TRA and DL-CIR. For the DL-REE with reentrant-shaped microchannels, given that the unique reentrant Ω-shaped microchannels featured narrow slots at the top and large circular cavities below, the abrupt protrusion between the circular cavity and vertical slot interrupted continuous flow streamlines in the cross section and exerted throttling effects on the fluid flow. This accelerated the main flow in the circular cavities [22] but slowed the flow in the narrow slot, as shown in Figure 13e. Large friction flow resistance was thus induced. Therefore, the DL-REE with reentrant microchannels also presented a large pressure drop. When comparing the DL-TRI with DL-REC, the DL-REC featured more corners than the DL-TRI. Moreover, the spanwise extension of the flow streamlines in the rectangular microchannels was restricted by its two vertical walls, as shown in Figure 13b. The flow friction resistance was thus enhanced, and a larger pressure drop can be noted for DL-REC. The DL-TRI with rectangular microchannels may reach a good balance without notable channel confinement in both vertical and spanwise directions, which would be helpful for the fluid flow with a small pressure drop.

**Figure 16:**
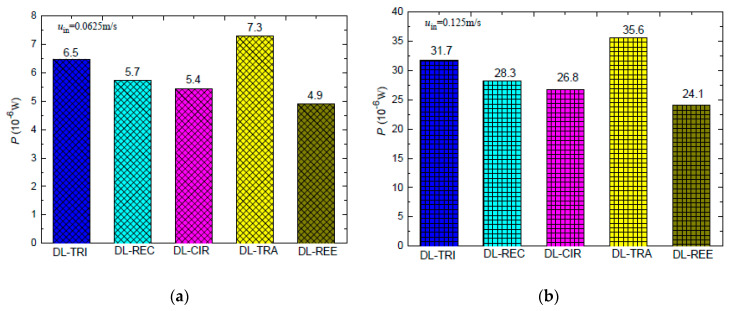
Pumping power of the five DL-MCHSs in the test cases of: (**a**) 0.0625 m/s; (**b**) 0.125 m/s.

When pumping power *P*, that is, the integration of pressure drop with cross-sectional area, is considered for the five DL-MCHSs with the same inlet velocity, it can be noted from Figure 16 that the DL-TRA with trapezoidal microchannels induced the largest pumping power consumption. For the other DL-MCHSs, the orders of *P* were just the opposite to those of the pressure drop, i.e., the smallest pumping power needed was for the DL-REE with reentrant microchannels, followed by DL-CIR, DL-REC, and DL-TRI. This trend, however, is consistent with the cross-sectional areas, as shown in Table 1. This indicates that cross-sectional area may play a more significant role in the total pumping power able to drive the fluid flow in double-layered microchannel heat sinks.

#### 4.2.3. Overall Performance Evaluation

When considering both thermal and hydraulic characteristics of the DL-MCHS, it can be noted that the DL-TRA with trapezoidal microchannels performed the worst in terms of thermal resistance, pressure drop, and pumping power. It should not be selected for double-layered microchannel heat sinks. On the other hand, the DL-REC with rectangular microchannels presented the smallest thermal resistance and the best wall temperature uniformity. It also presented small wall temperatures along the flow direction. As such, it showed the best overall thermal performance out of the five DL-MCHSs. The DL-TRI with triangular microchannels also showed fairly good thermal performance with low wall temperatures and small thermal resistance. In addition, these two DL-MCHSs showed small pressure drops. Nevertheless, when pumping power is considered, these two DL-MCHSs are no longer superior. The DL-REE with reentrant Ω-shaped microchannels exhibited the smallest pumping power. Therefore, the optimum DL-MCHS is dependent on which issue is considered the most important. When thermal performance is the prime concern, the DL-REC with rectangular microchannels seems to be the optimum choice. When pumping power consumption is the most important consideration, the DL-REE with reentrant Ω-shaped microchannels should be selected as a double-layered microchannel heat sink.

## 5. Conclusions

This work has presented a computational study of the thermal and hydraulic performance of double-layered microchannel heat sinks with different cross-sectional shapes. The superiority of double-layered microchannel heat sinks has also been assessed via a comparison of a single-layered and double-layered microchannel heat sink with triangular microchannels. Compared to the SL-TRI, the DL-TRI decreased wall temperatures considerably and presented a 27% reduction in thermal resistance. It also induced much more uniform wall temperature distribution, and significantly reduced the pressure drop and pumping power. For the five DL-MCHSs with different cross-sectional shapes, the DL-TRA with trapezoidal microchannels performed the worst in terms of thermal resistance, pressure drop, and pumping power. The DL-TRI with triangular microchannels and the DL-REC with rectangular microchannels presented small wall temperatures and thermal resistance, which are promising for maintaining high cooling efficiency. The DL-REC showed the most uniform temperature distribution and presented the best overall thermal performance. The DL-TRI and DL-REC also outperformed others in the reduction of pressure drop. Nevertheless, when pumping power is considered, the DL-REE with reentrant Ω-shaped microchannels performed the best. As such, the DL-REC with rectangular microchannels seems to be the optimum choice when thermal performance is the prime concern, whereas the DL-REE with reentrant Ω-shaped microchannels should be selected when pumping power consumption is considered to be the most important issue.

## Figures and Tables

**Table 1:** Specification of geometric parameters of the computational domain of microchannel heat sinks.

Sample	Arrangement and Cross-Sectional Shape of Microchannels	Width of the Unit Cell *W* (mm)	Height of the Unit Cell *H* (mm)	Length of the Unit Cell *L* (mm)	Thickness of Bottom Wall, *t_b_* (mm)	Hydraulic Diameter *D_h_* (mm)	Cross-Sectional Area of Microchannel *A_c_* (mm^2^)
DL-TRI	Double-layered triangular	2	4	45	0.79	0.81	0.849
DL-REC	Double-layered rectangular	2	4	45	0.8	0.8	0.72
DL-CIR	Double-layered circular	2	4	45	1.35	0.79	0.664
DL-TRA	Double-layered trapezoidal	2	4	45	1.33	0.8	0.77
DL-REE	Double-layered reentrant	2	4	45	0.93	0.76	0.587
SL-TRI	Single-layered triangular	2	2	45	0.79	0.81	0.849

**Table 2:** Temperature results for the five DL-MCHSs.

Sample	Layer	Inlet Velocity (m/s)	Maximum Temperature of Microchannel Base *T*_max_ (K)	Maximum Wall Temperature Difference Δ*T*_w,max_ (K)
DL-TRI	Upper	0.0625	344	7.2
	Lower			6.8
	Upper	0.125	332	5.4
	Lower			5.2
DL-REC	Upper	0.0625	342	5.6
	Lower			5.3
	Upper	0.125	328	3.9
	Lower			3.6
DL-CIR	Upper	0.0625	348	8.2
	Lower			8.1
	Upper	0.125	333	5.9
	Lower			5.8
DL-TRA	Upper	0.0625	356	8.0
	Lower			8.4
	Upper	0.125	339	5.6
	Lower			5.8
DL-REE	Upper	0.0625	350	5.9
	Lower			5.6
	Upper	0.125	333	4.2
	Lower			4
SL-TRI	-	0.125	358	24.3
